# The Fight against COVID-19 on the Multi-Protease Front and Surroundings: Could an Early Therapeutic Approach with Repositioning Drugs Prevent the Disease Severity?

**DOI:** 10.3390/biomedicines9070710

**Published:** 2021-06-23

**Authors:** Annamaria Vianello, Serena Del Turco, Serena Babboni, Beatrice Silvestrini, Rosetta Ragusa, Chiara Caselli, Luca Melani, Luca Fanucci, Giuseppina Basta

**Affiliations:** 1 Department of Information Engineering, Telemedicine Section, University of Pisa, 56122 Pisa, Italy; annavianelloa@libero.it (A.V.); luca.fanucci@unipi.it (L.F.); 2 Council of National Research (CNR), Institute of Clinical Physiology, 56124 Pisa, Italy; serena.babboni@ifc.cnr.it (S.B.); rosetta.ragusa@gmail.com (R.R.); chiara.caselli@ifc.cnr.it (C.C.); 3 Department of Surgical, Medical, Molecular Pathology, and Critical Area, University of Pisa, 56122 Pisa, Italy; beatrice.silvestrini@med.unipi.it; 4 Department of Territorial Medicine, ASL Toscana Nord-Ovest, 56121 Pisa, Italy; lucamelani@libero.it

**Keywords:** COVID-19, SARS-CoV-2, protease, ACE2, repositioning drugs, co-receptors

## Abstract

The interaction between the membrane spike (S) protein of severe acute respiratory syndrome coronavirus 2 (SARS-CoV-2) and the transmembrane angiotensin-converting enzyme 2 (ACE2) receptor of the human epithelial host cell is the first step of infection, which has a critical role for viral pathogenesis of the current coronavirus disease-2019 (COVID-19) pandemic. Following the binding between S1 subunit and ACE2 receptor, different serine proteases, including TMPRSS2 and furin, trigger and participate in the fusion of the viral envelope with the host cell membrane. On the basis of the high virulence and pathogenicity of SARS-CoV-2, other receptors have been found involved for viral binding and invasiveness of host cells. This review comprehensively discusses the mechanisms underlying the binding of SARS-CoV2 to ACE2 and putative alternative receptors, and the role of potential co-receptors and proteases in the early stages of SARS-CoV-2 infection. Given the short therapeutic time window within which to act to avoid the devastating evolution of the disease, we focused on potential therapeutic treatments—selected mainly among repurposing drugs—able to counteract the invasive front of proteases and mild inflammatory conditions, in order to prevent severe infection. Using existing approved drugs has the advantage of rapidly proceeding to clinical trials, low cost and, consequently, immediate and worldwide availability.

## 1. Introduction

Over the last two decades, there have been three deadly human outbreaks of coronaviruses (CoV), severe acute respiratory syndrome-CoV (SARS-CoV), Middle East Respiratory Syndrome-CoV (MERS-CoV), and SARS-CoV-2. The latter is causing the current pandemic called CoV disease 2019 (COVID-19). They target the human respiratory tract causing severe progressive pneumonia and could spread to other organs, causing damage to the central nervous system in SARS-CoV, severe renal failure in MERS-CoV, and multi-organ failure in SARS-CoV-2 [1].

Despite a high percentage of people with a positive screening test results asymptomatic or paucisymptomatic, COVID-19 can manifest as a respiratory tract infection with a serious spectrum of infection [2]. Severe symptoms, with hypoxia and pneumonia was reported in 15 to 20 percent of infections [3], with a critical associated acute respiratory distress syndrome (ARDS), which can rapidly progress to a multi-organ failure, irreversible and lethal in some cases [4,5]. Genomic studies confirmed the role of viral spike glycoprotein (S protein) in virulence and pathogenicity for SARS-CoV, MERS-CoV and SARS-CoV-2 [1].

The inflammatory cascade, fibrotic and coagulative events of COVID-19 start from the interaction between the membrane S protein of SARS-CoV-2 and the transmembrane angiotensin-converting enzyme 2 (ACE2) used as site of attachment to the host cell. However, its entry into the host cells is mediated by transmembrane proteases, of which the transmembrane serine protease 2 (TMPRSS2) is the main one. Recent studies have identified several key amino-acidic residues for S-protein interactions with the human ACE2 receptor and the TMPRSS2 membrane protease to initiate infection [6].

Although ACE2 is a target receptor for both SARS-CoV and SARS-CoV-2, the genetic variance observed in the homologous sequence of the gene encoding the S protein allows SARS-CoV-2 to bind efficiently to the receptor with firm attachment, improving virulence compared to SARS-CoV, and then causing very high morbidity and mortality worldwide.

Since ACE2 and TMPRSS2 are co-expressed in a limited number of tissues, the high viral transmissibility and the tissue tropism suggest that SARS-CoV-2 may use other proteases for cellular entry [7]. In fact, several proteases have been found to be involved in the transmission or infection process, including furin (a membrane-bound protease expressed in different tissues, mainly in the lungs [6]), ADAM17 (short for a disintegrin and metalloprotease 17), and cathepsin L.

Several studies have also focused on identifying additional mediators which may increase SARS-CoV-2 infectivity and contribute to the tissue/organ tropism. Some data are emerging for other cell mediators/receptors, including neuropilin-1 (NRP-1), integrins, sialic acids (SA), factor Xa, heparan sulfate (HS), cluster of differentiation 147 (CD147) and glucose-regulated protein 78 (GRP78) [8].

Given the complexity of interactions between viral proteins and host receptors with differing binding specificity and affinity, the differential prognosis for COVID-19 in SARS-CoV-2 positive patients may depend on the presence of single-nucleotide polymorphism in ACE2, serine proteases, mediators or co-receptors, either individually or combined with each other or even in combination with SARS-CoV-2 genetic variants resulting in more or less virulent and lethal strains [9].

To date, effective antivirals for counteract COVID-19 have not been found and many bio-molecular mechanisms of SARS-CoV-2 infection remain elusive.

The identification of key factors, such as receptors and proteases, involved in the dynamic of infection, could provide a way to stop the spread of the virus and suggest single or combined therapeutic treatments to counteract host binding and multi-protease activation.

Since there is a short therapeutic time window in which the rapid progression of the disease does not yet exceed the therapeutic potential of the available drugs, we believe that an early pharmacological approach would be extremely useful.

We review some questions regarding the first stage of virus–host interaction: What are the mechanisms by which cells are infected, and what existing or potential drugs can interfere with them? We explore the early molecular processes of SARS-CoV-2 infection by highlighting the new therapeutic insights that have emerged from the various basic and clinical research studies currently underway. We also discuss the possibility of using off-label drugs, which may have benefit of limiting the progressive spread of SARS-CoV-2 in infected individuals.

### Literature Search Strategy

We performed a systematic literature search using PubMed, SCOPUS, Web of Science, Google Scholar, and MedRxiv/BioRxiv (preprints) databases. We included scientific publications and preprint articles from 1 January 2020 to 30 April 2021 and books, book chapters, conference abstracts were excluded. All searches and study selection were performed by three investigators working independently. The search queries were COVID-19 AND “cell-entry”, “viral fusion”, “protease-inhibitor”, “repurposing-drug”. The search was also repeated by using the above-mentioned keywords and the term “SARS-CoV-2” instead of “COVID-19”.

Between the initial 574 studies that were collected through the electronic search, 49 were excluded because of the duplicated results, 133 were omitted due to non-original study, 155 review papers were ruled out while some reviews considered useful for the introduction of some topics were included, and 94 were considered unrelated based on abstract and/or title data because focused on antiviral, immunomodulators, vaccines, and other non-early treatments. In addition, 10 were omitted as they were not in the English language. Finally, 133 papers were included in this systematic review following the literature search and selection process. All articles judged as potentially eligible were retrieved for full-text review. We screened all reference list of the most pertinent studies in order to identify any missing publications.

## 2. The Binding of SARS-CoV-2 to ACE2 Receptor Is Just the Tip of the Iceberg: The Submerged Front of Multi-Proteases

Both SARS-CoV and SARS-CoV-2 utilize ACE2 as a host-cell entry receptor and proteases as entry activators [10] (Table 1). ACE2 is a negative regulator of the renin–angiotensin system (RAS), which is essential for maintaining blood pressure homeostasis and the balance of salts and fluid. This regulation is critical for the physiopathology of various organs, including lungs, kidneys, and heart. ACE2 also regulates the absorption of amino acids in the gut and kidney, then modulating the expression of transporters for amino acids [10,11].

ACE2 contains a single catalytic domain with zinc-binding motif located at the extracellular side of the cell, which can be cleaved and released into the bloodstream by ADAM17 [12].

COVID-19 patients exhibit multi-organ dysfunction, due to the expression of ACE2 in the lung, heart, vascular system (endothelial cells and smooth muscle cells), brain, kidney, gut and testis [29,30]. Particularly, 80% of all ACE2-expressing cells were identified in type II pneumocyte cells, followed by the nasal and oral mucosa and alveolar macrophages [31]. ACE2 is also expressed in pericytes—undifferentiated and contractile cells that surround the capillary endothelial cells particularly in renal circulatory plexus—and in cardiac myocytes. This latter would explain the high renal microvascular damage and heart failure incidence in COVID-19 [32].

**Figure 1:**
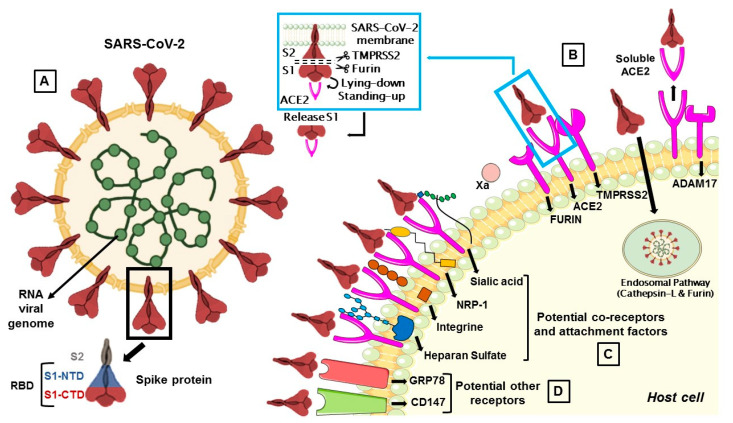
(**A**) The membrane spike protein of SARS-CoV-2 consists of three glycoprotein monomers, and each monomer contains two subunits, S1 and S2. The S1 subunit is divided into the N-terminal (NTD) and C-terminal (CTD) domain, which is referred to as the receptor-binding domain (RBD), while the S2 subunit contains membrane fusion activity. (**B**) SARS-CoV-2 entry mediated by TMPRSS2 and cathepsin-L. Once the S protein interacts with ACE2, TMPRSS2 cleaves S protein at the S1/S2 cleavage site and then the furin cleaves the S2 region (S2’) to initiate conformational changes for membrane fusion. The S protein can also activate ADAM17 which can cleave ACE2, resulting in shedding and soluble ACE2. In a second route, the S protein is cleaved and activated by the cathepsin-L pathway, where virions are taken up into endosomes. (**C**) Co-receptors are involved in SARS-CoV-2 attack. S proteins can recognize sialic acids, NRP-1, integrins and heparan sulfate for the first step of attachment to host cells. (**D**) CD147 and GRP78 have been identified as other potential receptors for SARS-CoV-2 entry.

The S protein, by which SARS-CoV-2 recognizes the host ACE2 receptor, consists of three glycoprotein monomers and each monomer comprises two subunits, S1 and S2. S1 subunit can be further divided into an N-terminal domain (NTD) and a C-terminal domain (CTD). This latter domain is referred to as the ACE2 receptor-binding domain (RBD) for SARS-CoV-2 (Figure 1A) [14,16]. S1 RBD is the most variable part of the SARS-CoV-2 genome and key residue substitutions in this region can enhance the interaction ability and lead to a higher binding affinity between the S protein and ACE2 [33]. Genetic variants of SARS-CoV-2 have been emerging and circulating around the world and are frequently monitored through sequence-based surveillance, laboratory studies, and epidemiological investigations [34,35].

There are two proteolytic activation events associated with the virus–cell membrane fusion process, mediated by the S protein, which can involve several proteases [14,15,36].

The first is a priming cleavage at the interface of the S1 and S2 subunit, which allows both to expose hidden cleavage sites and to increase binding affinity for the receptor. The second is a triggering cleavage that occurs within the S2 region (S2′) and allows the liberation and the conformational changes of S2 fusion peptide that mediate the fusion of the viral envelope with the host cell membrane [37].

The protease activity of ACE2 has no role in facilitating viral entry and it merely appears to act as a receptor to guide attachment and fusion of the S protein. In fact, following the binding between S1 subunit and ACE2 receptor, different serine proteases, such as TMPRSS2, cathepsin-L, and the metalloproteinase ADAM17, trigger the cleavage of ACE2 and the consequent fusion of the viral membrane to the host. Differently from SARS-CoV, the S protein of SARS-CoV-2 is activated by the serine protease furin, which facilitates conformational changes required for S1 RBD exposure and binding to surface receptors (Figure 1B) [38].

S protein has a closed and an open conformation: in the closed form, all three RBDs of the S trimer are inaccessible to ACE2 binding, while in the opened form, RBDs are exposed to ACE2 binding. After furin cleavage between the S1 and S2 domains (priming), the proportion of the S trimers in the open conformation increases. The final link between RBD and ACE2 leads to a fully open form, in which the S1 subunit remains limitedly bound to the core S2 trimer through intermediate S1 subdomains. This arrangement leaves the top of the S2 fully exposed for the next fusion of the membranes [14].

### 2.1. Furin Priming

Furin is a Ca^2+^-dependent endopeptidase, responsible for activating precursor proteins like fusion proteins of viruses. Furin is capable of cleaving and activating viral fusion proteins of HIV, Ebola [39], MERS-CoV [40], SARS-CoV [15], and SARS-CoV-2) [16] (Table 1). Furin accumulates mainly in the Golgi complex, but it can be transported to the cell surface via the endosomal pathway or can be released into the extracellular space [41]. For this reason, furin can cleave the S protein in the Golgi complex, and also in the extracellular space [38].

The addition of a furin cleavage site to S1/S2 is essential for efficient viral entry into human lung cells, and its presence expands the versatility, transmissibility and tropism of SARS-CoV-2 due to the wide cellular expression of furin proteases [42,43].

Many health conditions, like diabetes, obesity and hypertension, are associated with elevated furin levels, and this circumstance can explain why patients with such pre-existent comorbidities are subject to severe forms of COVID-19 [44]. High levels of furin suggest testing for potential furin inhibitors to counteract COVID-19. Some of them, such as the furin convertase inhibitor (chloromethyl ketone) and peptidyl chloromethyl ketones have already been reported for HIV [45], but not yet evaluated for SARS-CoV-2. There are also ongoing studies that use phytochemicals, like bromhexine and phyto-flavonoid luteolin, which are validated to block the S-protein cleavage activation and membrane fusion [46] (Table 2). Another potential candidate for adjuvant treatment is the trypsin inhibitor tamarind, which in addition to several beneficial effects on the reduction of inflammatory markers, could inhibit alone or in combination with other drugs the action of proteases that facilitate the SARS-CoV-2 infection [47].

### 2.2. TMPRSS2

TMPRSS2 is a multi-domain type II transmembrane serine protease, that mainly cleaves the S protein of SARS-CoV, MERS-CoV and SARS-CoV-2 to trigger fusion of viral envelope with the host cell membrane (Table 1). It is also important for the entry of the influenza virus into the host cell [1]. TMPRSS2 plays a dual role in the infection process: it proteolytically cleaves both the S protein, to trigger fusion of viral envelope with the host cell membrane, and the ACE2 tail, to increase the virion uptake, also through the cathepsin L -dependent pathway [68] (Figure 1).

TMPRSS2 and ACE2 are co-expressed in the lung, heart, gut, smooth muscle, liver, kidney, neurons, and immune cells [68,69,70,71]. In particular, they are both observed within the type II pneumocyte cells [72]. The TMPRSS2 gene is also expressed in the adult prostate, as reported in a research in which men were at higher risk for developing the disease with severe symptoms [73]. Indeed, the possible role of androgen receptors in increasing SARS-CoV-2 infection through the regulation of TMPRSS2 transcription has recently emerged, suggesting crosstalk between COVID-19 and prostate cancer, caused by the elevated expression of TMPRSS2 [74].

The exact sequence of cleavage events is not yet clear, but it appears that first furin cleaves at S1/S2 domain and then TMPRSS2 cleaves at the S2 cleavage site to produce fusion peptide and S2′ to trigger membrane fusion (Figure 1B) [16]. However, TMPRSS2 plays a dual role during the infection process. Beyond the afore-mentioned cleavage of the S protein, it proteolytically cleaves the ACE2 tail that event promotes the uptake of virions through the cathepsin L-dependent pathway [68].

Thus, there are two pathways for SARS-CoV-2 entry into the host cells either by endocytosis, which leads to the fusion of the viral membrane with the host cell membrane or via endosomal mechanism activated by cathepsins [75].

Given the crucial role of TMPRSS2 in favouring viral entry and the absence of approved therapies for treating the ongoing pandemic, initial attention has focused on drug repurposing opportunities to inhibit this protease (Table 2). TMPRSS2 is a protein that can be inhibited by camostat, nafamostat, and gabexate, clinically approved chemical agents in Japan for the treatment of pancreatitis [48]. There are currently several clinical trials listed on “https://clinicaltrials.gov/” to investigate the use of these repurposing drugs against SARS-CoV-2. Some data suggest that nafamostat and camostat have the potential to block S-protein cleavage mediated not only by TMPRSS2 but also by other proteases [49].

A more recent study has highlighted the correlation between a TMPRSS2 variant with a high number of cases and/or deaths of COVID-19 observed in different countries [76]. This epidemiological evidence strengthens the usefulness of TMPRSS2 inhibitors for COVID-19 management.

An in silico molecular docking study was recently performed targeting potential phytochemicals and drugs that prevent the entry of SARS-CoV-2 into host cells by inhibiting the proteolytic activity of furin and TMPRSS2 [13]. Among these, the drug nafamostat may be more beneficial than camostat in suppressing the activity of TMPRSS2. Among the phytochemicals, polyphenols in green tea were found also be potentially useful in suppressing the furin activity [13]. Limonin and gedunin found mainly in the citrus fruits and neem showed the highest binding energy at the active site of furin and TMPRSS2, respectively [13].

The activation of TMPRSS2 and other proteases also facilitates cell–cell fusion mediated by SARS-COV-2, leading to the formation of multinucleated cells, called syncytia, which contribute to tissue damage [77]. It has recently been reported that high concentrations of bromhexine, an expectorant and inhibitor of TMPRSS2 currently used in clinical trials against COVID-19 [78,79], unlike another expectorant, ambroxol, may favor the formation of syncytia [80]. These results suggest greater caution in the use of high-dose bromhexine until its effects on COVID-19 are fully understood.

From a screening of more than 3000 existing approved drugs, about 83 drugs were shown to be efficient for inhibition of S-protein-mediated cell fusion [51]. By focusing on effective drugs that also protect against virus replication and associated cytopathy, one of the most effective drugs was the anthelmintic drug niclosamide, suppressing the activity of TMEM16F (also known as anoctamin 6), a calcium-activated ion channel involved in syncytia formation [51]. In addition, it seems likely that, similar to niclosamide, all drugs, as trifluoperazine [81], serotonin reuptake inhibitors [82,83] and ivermectin [52] (Table 2), that inhibit TMEM16 proteins, block SARS-CoV-2 S-protein-induced syncytia.

### 2.3. Cathepsin L-Dependent P-Pathway

Cathepsins are cysteine proteases with a crucial role in protein catabolism in the endosomes and lysosomes. They require low pH (between 4.5–5.0) for optimal proteolytic activity of protein antigens resulting from pathogen endocytosis [84].

Cathepsin L is ubiquitously expressed in mammalian cells and seems to be important for SARS-CoV-2 entry in human cells as a result of cleaving the S2′ position and activate the fusion between virus and endosomal membrane, leading to the release of the viral genome into the host cells (Figure 1B) [85,86].

Drug repurposing efforts for SARS-CoV-2 have targeted endosomal cathepsins [87]. Some studies have suggested the use of the chloroquine, a broadly used antimalarial drug, and of its derivative hydroxychloroquine for COVID-19 treatment [53,54,55,88]. Chloroquine inhibited the SARS-CoV-2, SARS-CoV, influenza virus, Ebola and other viruses in vitro, perhaps through interference with the endocytic pathway [89]. However, until now, its clinical effectiveness is limited in COVID-19 patients and its potential cardiac side effects are the main concerns [53,54,55,88].

The cross-linking peptide, named 8P9R, can be another potential interferent with the endocytic pathway. Beyond its direct action of virus cross-linking activity, it could reduce the endosomal acidification inhibiting the viral entry [90]. The combined use of two endosomal acidification inhibitors (8P9R and chloroquine) improved the antiviral efficiency of the drug umifenovir, a S-protein–ACE2 fusion inhibitor, clinically available for prophylaxis and treatment of influenza in China and Russia, and now in Phase III and IV clinical trials in the USA [91] (Table 2). Since ACE2 and TMPRSS2 are individually or co-expressed in human cells, the approach of simultaneous inhibition of virus entry through blockage of both endosomal and surface fusion pathways may have better antiviral results.

### 2.4. ADAM17 and Soluble ACE2

The type-I transmembrane protease ADAM17 is expressed in many tissues, including lungs, muscles, heart, kidney, small intestine, pancreas, placenta, ovaries and testicles, and is involved in ectodomain shedding of cell membrane proteins [92].

The viral S protein can activate ADAM17 to cleave ACE2, resulting in a soluble ACE2 (sACE2) shedding and release (Figure 1B). How ADAM17 facilitates viral entry is not yet clear, but it might contribute to the fusion of viral particles with the host cell membrane [70].

The involvement of ADAM17 in the SARS-CoV-2 cell entry is still unclear, although it is involved in the RAS imbalance associated with virus infection [17].

According to Zoufaly et al. [93], the administration of recombinant sACE2 could act through two mechanisms either by binding to S protein and neutralizing the viral particles and the other by increasing the concentration of angiotensin II, thus helping to reduce multi-organ damage. In addition, Monteil et al. [94] showed that the administration of recombinant soluble ACE2 along with the antiviral remdesivir has an additive effect at sub-toxic concentrations and may improve the effect of remdesivir during SARS-CoV-2 infection.

## 3. Co-Receptors for ACE2: Sialic Acids, Neuropilin-1, Heparan Sulfate and Integrins

In addition to ACE2, also sialic acids (SA) [95], NRP-1 [96], integrins [97], and heparan sulfate (HS) [25], have been identified as other possible cell surface attachment mediators for SARS-CoV-2. Since the research in this field is very intense and given the need to find intervention strategies to block host/virus interaction, the number of potential mediators may rapidly increase.

### 3.1. Sialic Acids

The S protein is a lectin-like protein that recognizes O-acetyl sialic acids (O-Ac-SAs), during the attachment to host cells. O-acetylation is a common sialic acid modification that facilitates the interaction with viruses, antibodies and lectins (Figure 1C) [98].

Many betacoronavirus are able to bind with sugar-like receptors through NTD domain of S1 subunit [21]. In particular, they bind O-Ac-SAs and use a hemagglutinin-acetyl-esterase glycoprotein receptor to mediate the viral attachment. The S protein recognizes the 9-O-Ac-SA sugar, while the hemagglutinin-acetyl-esterase acts to release virions from infected host cells [88].

Binding to sialic acids may help the virus pass through the mucus layer, which is rich of SA and covers the viral target cells in the epithelium of the small intestine [99]. The potential mechanism involves the binding of the S1 subunit both to ACE2 receptor and ganglioside domain (sialic acid-containing glycosphingolipids) of the gut plasma membrane by RBD and NTD, respectively, favoring the subsequent interaction of the RBD with ACE2 [71,100,101].

Another study instead identified that sialic acids present on ACE2 as deterrent for SARS-CoV-2 infection, because they inhibit efficient S protein/ACE2-interaction [95]. Sialic acids were more of an obstacle for SARS-CoV infection than for SARS-CoV-2 infection [95]. Further studies are required to give evidence that that sialic acids play a critical role in SARS-CoV-2 attachment [102].

### 3.2. Neuropilin-1

NRP-1 is a membrane-bound co-receptor to a tyrosine kinase receptor for both vascular endothelial growth factor and semaphorin family members. Recently, NRP-1 has been identified as a possible novel factor for SARS-CoV-2 host cell entry [103]. After cleaving by furin, S1 and S2 proteins are exposed. In particular, when S1 is exposed, the C-terminal domain containing a cationic amino acid, such as arginine, binds to the b1 domain of NRP-1 and potentiates virus infectivity (Figure 1C) [22]. It has been demonstrated that the co-expression of NRP1 with ACE2 and TMPRSS2 markedly enhances the infection of SARS-CoV2 [104]. However, it is still unclear whether Nrp1 may be a co-binding factor together with ACE2 and whether they interact directly or indirectly. NRP-1 is expressed in all tissues, including endothelial cells, vascular smooth muscle cells, lung cells, macrophages, adipose tissue, retinal vasculature and neurons [96,105]. A number of studies reported overexpressed levels of NRP-1 in COVID-19 patients [104,106]. Further evidence shows that the virus invades the olfactory epithelium, which is full of cells carrying NRP-1, and this could explain the early loss of odor perception in infected patients [107].

Perez-Miller et al. [57] described that from an in-silico screening of nearly 0.5 M of compounds, nine chemical series (natural products and small molecules) targeted the C-end rule peptide at the binding site on NRP-1. The interaction between NRP-1 and HS could facilitate the interaction of the S protein with NRP-1, which highlights the speculation of a potential synergistic effect of heparin (a polysaccharide structurally similar to HS and largely used for hospitalized SARS-CoV-2 patients) and NRP-1 inhibitors as a drug combination to prevent the viral entry.

### 3.3. Heparan Sulfate

Recent studies identified HS as a co-receptor for SARS-CoV-2 entry [26]. HS polysaccharides are ubiquitous components of the cell surface and extracellular matrix, with multiple functions, such as cell signalling, growth, adhesion, migration and host–pathogen interactions [26]. The S protein interacts with HS and this binding shifts the structure to favor the RBD open conformation that binds ACE2. Interestingly, HS is able to bind to RBD in both open and closed conformation, while ACE2 can bind to RBD only in an open conformation (Figure 1C) [25]. HS can also interact with antithrombin, a member of the serine protease inhibitor superfamily and critical anticoagulant regulator, causing a conformational change that results in an active form of antithrombin [108]. This could have serious consequences in the cardiac district since the heart has a high expression of ACE2 that facilitates the interaction between SARS-CoV-2 and HS [109]. Consequently, the HS consumption leads to lower levels of antithrombin, creating a hypercoagulable state, endothelial injury and intracardiac thrombus formation characterizing in COVID-19 patients [110]. COVID-19 patients with hyper-coagulopathy showed a better prognosis in relation to mortality with heparin or low-molecular-weight heparin treatment [111,112,113]. HS shares high structural similarity with heparin, but their functionalities are differentiated by the interactions with various proteins, such as proteases and protease inhibitors, cytokines and their respective receptors [114]. Recently, the preclusion of interaction between HS and S protein from heparin/HS mimetics has been proposed like anti-SARS-CoV-2 drugs [58]. Furthermore, Tandon et al. [59] also suggested that intranasal administration of unfractionated heparin could be used as a prophylactic treatment of SARS-CoV-2.

### 3.4. Integrins

The RBD within S1 subunit S1 subunit contains a conserved RGD (arginine-glycine-aspartate) motif, known to bind integrins [97]. This minimal peptide sequence required to bind with integrins is common for some viruses like Rotavirus, cytomegalovirus, Epstein–Barr virus and Ebola, but not in SARS-CoV [23]. Integrins are cell-surface transmembrane receptors responsible for platelet aggregation, cell adhesion, cell migration, and cell-signalling processes [23]. Since the RGD motif is close to the ACE2 receptor-binding region of the S1 subunit, integrin may act as co-receptor that could increase the binding potency of ACE2 for SARS-CoV-2 (Figure 1C), explaining the fast spread and aggressiveness of the virus [115]. On the other hand, integrin may act as an alternative receptor for SARS-CoV-2 and could expand cell tropism and potentially affect the viral pathogenicity and spread.

Furthermore, the binding between ACE2 and the S-protein induces conformational changes into binding subdomains, allowing the RGD exposure [24]. The mutations, recently seen in the United Kingdom and South African variants, led to a greater surface exposure of the RGD domain resulting in a higher binding with integrins and a higher transmissibility than the Wuhan strain [97]. Evidence showed that the RGD domain of SARS-CoV-2 could have a unique sequence in the proximity of ACE2 binding region, so another therapeutic option could be RGD mimetics used as inhibitors of virus attachment [116]. ATN-161, an integrin-binding peptide, has proven to inhibit the interaction between the S protein and integrins, by competing with RGD domain. ATN-161 represents a promising therapeutic approach and could be rapidly introduced into clinical trials [60].

## 4. Other Receptors for SARS-CoV-2 Entry: CD147 and GRP78

### 4.1. CD147

Recently CD147, also known as basigin or extracellular matrix metalloproteinase inducer, has been identified as another possible receptor that mediates viral entry (Figure 1D) [27].

CD147 is a mediator of inflammatory and immune responses and is involved in tumour development, plasmodium invasion and virus infections [117]. CD147 is expressed on leukocytes, platelets, endothelial cells and in the lung, type II pneumocytes and the macrophages at the edges of the fibrotic zones caused by COVID-19 [61].

The role of CD147 as cell entry receptor during SARS-CoV-2 infection is still not clear. However, Wang et al. [27] showed a direct interaction between the S-protein RBD and CD147 in vitro, suggesting that the inhibition of CD147 could have a role for pulmonary fibrosis resolution in COVID-19 patients [118].

CD147 was also identified as a red blood cell receptor for the parasite Plasmodium falciparum—protozoan (PfRH5)—which causes Malaria in humans [119]. Azithromycin, an antibiotic widely used to treat chest infections that destroy parasites and also acts as an inhibitor of red blood cell invasion, preventing the essential step of tight junction formation [119]. Azithromycin can interfere with the PfRH5/CD147 interaction, so might represent a novel therapeutic approach against other pathogens such as SARS-CoV-2 that invade cells by binding to CD147 [61].

CD147 can interact with cyclophilin A, a cytosolic protein that binds to the immunosuppressant cyclosporin A, suggesting the use of cyclosporine A to inhibit the entry of SARS-CoV-2 into host cells [62].

### 4.2. GRP78

GRP78, a heat shock protein A5 or binding immunoglobulin protein, has a site of interaction with the RBD of the S protein, suggesting a possible role for the viral pathogen internalization (Figure 1D) [28,120]. Patients infected with SARS-CoV-2 have high gene expression and serum concentrations of GRP78 [121].

A recent study showed an increase in the circulating levels of GRP78 in COVID-19 patients compared to patients with pneumonia or controls [121]. In the lung tissue of ARDS patients, endothelial barrier dysfunction occurs with consequent circulating increase of heat shock proteins. Therefore, high circulating levels of GRP78 in COVID-19 patients could derive from damaged airway epithelial cells [17,121].

From an in-silico screening of available databases of bioactive peptides and polyphenolic compounds against the S protein, the binding site in GRP78, and with the ATPase domain GRP78, four small molecules have been identified: epigallocatechin gallate, omoeriodictyol, isorhamnetin, and curcumin [65]. Another study showed that phytochemical compounds, particularly Berbamine (Berberis vulgaris), have a greater inhibitory effect on GRP78 than the drugs OSU-03012 and epigallocatechin-3-gallate [66] (Table 2). Although polyphenols have anti-inflammatory and antiviral properties that may be useful for viral prevention and treatment, clinical studies are needed to demonstrate their potential efficacy against SARS-CoV-2 infection.

## 5. Effectiveness of Anti-inflammatory, Antibiotic and Anti-coagulant Treatments in the Early Stage of Infection

Anti-inflammatory and anti-coagulative therapeutic strategies used to control cytokine storm, endothelitis and thrombosis—clinical manifestations of later phases of disease—have been shown to be effective even at the early stage of the infection, regardless of inhibiting the binding of SARS-CoV-2 to receptor on host cell (Table 2).

### 5.1. Usefulness of Anti-inflammatory Drugs Administered Upstream of Hyperinflammation

The host innate immune response is the first line of defence against viral particles (RNA and viral proteins) and is usually coordinated by IFN-type cytokines that activate cells and intensify the response against these invading agents [122].

However, the immune system may play a dual role in SARS-CoV-2 infection. In more than 85% of cases, a proportionate immune response helps eliminate the virus, and the patient become asymptomatic or pauci-symptomatic. However, in only 10–15% of cases, the patient’s immune response is too intense and disproportionate. An immuno-pathological phase then follows the viral invasion and the patient develops a severe form of the disease [123].

The excessive inflammation in response to viral infection is responsible for severe forms of the disease, characterized by hypoxemic pneumonia, up to associated acute respiratory distress syndrome, sometimes associated with multi-organ failure. This highlights the need for a “right balance” of immune responses to infection: if there is a deficit, the infection spreads, but if there is an excessive response, hyper-inflammation (called “cytokine storm”) organ damage can occur [124]. Pro-inflammatory cytokines are produced by cells of the innate immune system (monocytes/macrophages/polymorphonuclear cells), which recognizes the pathogen-associated molecular patterns (PAMP) (viral proteins and nucleic acids), through pattern recognition receptors, (PRR), such as Toll-like receptors -7 and -8 [125,126,127]. The interaction between PAMP and PRR induces an intracellular signaling cascade at the origin the expression of pro-inflammatory cytokines, such as interleukin-6 (IL-6), tumor necrosis factor-α or IL-1β [128]. IL-6 is synthesized by immune cells but also by other types of cells, such as endothelial cells. IL-6 activates innate immunity cells, as well as adaptive immunity cells, in particular lymphocytes (T helper [Th] 17) and Th follicular cells [123].

Some immunotherapies targeted directly to the cytokine storm have been evaluated in COVID-19 patients [129,130]. However, it has been evident that an optimal benefit of anti-cytokine therapies may be plausible if administered during a restricted window of time, at the onset of “cytokine storm” but before sudden disease causes irreparable tissue injury [131,132].

Corticosteroids exert their anti-inflammatory effects mainly by transcription inhibition of genes encoding proinflammatory cytokines, chemokines, inflammatory enzymes to control the inflammatory process and restore homeostasis. The inhibition of the aberrant inflammation through timely administration of glucocorticoids in the early stage of an inflammatory cytokine storm may efficiently inhibit ARDS onset and preserve the organs’ functions [133,134].

The utility of corticosteroids emerged from the observation that patients with chronic respiratory disease, who make widespread use of inhaled glucocorticoids, were significantly underrepresented among those admitted to hospital with COVID-19 [135]. In a recent randomized controlled trial [136], the early administration (within seven days of the onset of mild symptoms) of inhaled budesonide, reduced the likelihood of needing urgent medical attention and reduced recovery time in adults after early COVID-19. An interesting observational matched-cohort study showed that early home treatment of 90 consecutive patients with mild COVID-19 by their family physicians according to a pathophysiologic and pharmacologic rationale almost completely prevented the need for hospital admission, due to progression toward more severe illness, compared to 90 age-, sex-, and comorbidities-matched patients who received other therapeutic treatments [67].

The key points of this successful therapeutic recommendation were: early intervention at the onset of mild/moderate symptoms at home, with specific non-steroidal anti-inflammatory drugs (mainly cyclo-oxygenase-2 inhibitors) [67]. Cyclo-oxygenase-2 has a significant effect on pro-inflammatory cytokine cascade induction and its inhibition does not attenuate the immune response against viral diseases [67]. COX-2 is responsible for producing most of the prostaglandins responsible for pain and inflammation [44]. In particular, it has been suggested that prostaglandin E2 (PGE2) may have a significant role in the hyper-inflammatory and immune response of COVID-19 patients [67]. Therefore, lowering PGE2 levels through inhibition of human microsomal prostaglandin E synthase-1 (mPGES-1) could improve the host immune response against COVID-19 and could be a promising therapeutic strategy to prevent progression of severe illness and death.

### 5.2. Usefulness of Antibiotics Administered Upstream of Hyperinflammation

Tetracycline and its derivatives (e.g., doxycycline and minocycline) are non-traditional antibiotics that have antiprotease properties. Their efficacy has been demonstrated against viral pathogens such as dengue fever and chikungunya [137]. Their well-established safety profile places them as possible drug candidates at all stages of SARS-CoV-2 infection, from replication to systemic response. The antiprotease properties of tetracyclines give them an advantage to improve the response to viral infection [138]. These pleiotropic characteristics of tetracyclines (anti-inflammatory and antiviral activities) are likely derived from the actions on different and little-known molecular pathways of non-traditional antibiotics [139].

Doxycycline has several potential mechanisms of action through which it may prevent or ameliorate the effects of COVID-19 infection [63,64]. Structural analysis demonstrated that doxycycline has the potential to inhibit two viral proteins both essential to viral replication and lifecycle: papain-like proteinase and 3C-like main protease. Doxycycline is well-known to inhibit IL-6 and metalloproteinases, in particular metalloproteinase-9, which is likely required for initial viral entry into the cell [63]. Low-dose doxycycline inhibited the expression of CD147, which may be used for SARS-CoV-2 entry into lymphocytes [64,140].

### 5.3. Coagulation Factors and an Exacerbate Production of Antibodies Enhance Viral Entry

Considering that pathogenesis of SARS-CoV-2 infection is associated with coagulopathy and thromboembolic events, circulating proteases, involved in blood clotting, can contribute to S-protein cleavage activation and enhance the viral entry [19].

While early-stage disease of COVID-19 is typically limited to local pulmonary hypercoagulability, severe late-stage disease may be accompanied by systemic disseminated intravascular coagulation [141], stroke, and cardio-embolism [142,143]. Precise molecular mechanisms related to SARS-CoV-2 infection and dysregulation of haemostasis are not yet clear, but several plausible suppositions have been hypothesized, given the strong relationship between inflammation and haemostasis. Acute lung injury from viral cytopathic effects, the induction of the COVID-19-associated cytokine storm, complement activation, and anti-phospholipid autoantibodies have all been suggested to prompt the coagulation cascade [144].

The clotting cascade is propagated by a chain reaction of serine proteases, including factor Xa and thrombin that are each activated by proteolytic processing [145]. Recently, it has been demonstrated that factor Xa and thrombin are able to directly cleave SARS-CoV-2 S-protein, leading to enhancing the viral entry [20].

Therefore, the possibility of a positive feedback was hypothesized in which infection-induced hypercoagulation increases the SARS-CoV-2 infectivity through a massive increase in viral entry facilitated by a multi-protease action [146].

Therefore, coagulation activation can exacerbate SARS-CoV-2 infectivity in both TMPRSS2 positive and TMPRSS2 negative host cells and can ubiquitously promote virus entry into all cell types and regions of the airways. It remains to be ascertained whether hypercoagulation in the early stages of COVID-19 is also linked to an extrapulmonary infection, such as the central nervous system [147] and small intestine [148].

The excessive production of antibodies released during an aberrant inflammatory response, can represent another possible mechanism for SARS-COV-2 entry. Wang et al. [149] revealed that the anti-S-protein antibodies were responsible for the in vitro infection of immune cells, and the enhancement of the infection can be improved by increasing the dilutions of antibodies. During the binding of the virus-antibody complex [S protein with antigen-binding fragment regions (Fab) of immunoglobulin G], the fragment crystallizable (Fc) portion of the antibody simultaneously binds to Fc gamma receptors expressed by immune cells, promoting viral entry without the use of the ACE2 receptor [150].

Effective anticoagulation is a critical area of investigation to improve the outcomes in coronavirus infection [29,151,152]. Vitamin K antagonists, including heparin, are commonly used to prevent venous thromboembolism in COVID-19 patients, although there is no strong evidence to support any specific anticoagulant [153]. Retrospective analysis suggested that low molecular-weight heparin could be beneficial for patients with signs of coagulopathy [113].

However, three large randomized clinical trials to determine the benefit of therapeutic-intensity vs. prophylactic intensity of heparin in critically ill COVID-19 patients were suspended at interim analysis for futility (NCT02735707, NCT04505774, and NCT04372589 in clinicaltrials.gov). Also, results of moderately ill hospitalized patients are pending [154,155]. Optimal protocols for managing coagulopathy in COVID-19 patients have not yet been developed.

A screening of existing drugs identified a subset of protease inhibitors that equally inhibited S-protein cleavage by both transmembrane serine proteases and clotting factors [13,19]. For example, the action of nafamostat and camostat can go beyond the inhibition of TMPRSS2, because they can also act on coagulation factors [19]. Anticoagulation is critical in the management of COVID-19, and early intervention could provide a side benefit by suppressing the entry of SARS-CoV-2. Nafamostat has also been used as an anticoagulant during haemodialysis [156] and extracorporeal membrane oxygenation [157], and to manage disseminated intravascular coagulopathy [158]. Inhibition of coagulation factor-induced S-protein cleavage may contribute to the molecular mechanism of these agents, if treatment is given sufficiently early.

α-1-antitrypsin is a serine protease inhibitor and constitutive tissue protector that can inhibit TMPRSS-2 and ADAM17 [50]. The inhibition of ADAM17 reduces the Fc gamma-receptor release from neutrophils, diminishes cell migration during inflammation and prevents ACE2 shedding. Hence it may preserve ACE2 inhibition of bradykinin, reducing the ability of bradykinin to cause a capillary leak in COVID-19 [50].

The oral anticoagulant serine protease inhibitors, otamixaban and dabigatran, have shown off-target activity against TMPRSS2 and other proteases, albeit at higher than safe concentrations used in vivo [159,160].

In summary, early intervention with oral anticoagulants has a strong potential to inhibit coagulation-factor-induced S-protein cleavage as well as managing coagulopathy and thrombotic complications.

## 6. Conclusions and Prospective

The binding of SARS-CoV-2 to the ACE2 receptor is just the tip of the iceberg. In fact, the multi-protease front is extremely complex and the chain reaction activation for different proteases takes place according to a cascade mechanism and an exponential kinetic [7,12,19,88,161].

The front of the multi-protease cascade is closely interacting with each other and their “storm effect” greatly increases the high risk of therapeutic failure over time [50,72,139].

Unfortunately, all new pharmaceutical agents, resulting from the most sophisticated molecular engineering technologies, will require a long time for preclinical evaluations before entering clinical practice. Their pharmacological effects on the delicate and complex balance of the multi-protease system require advanced studies to evaluate the pharmacokinetic and pharmacodynamic properties, and the precise timing of administration. Furthermore, the expected pharmacological effects could be neglected or even harmful. Thus, “old drugs”, in addition to their new and improved indications for COVID-19 patients, could represent useful clinical models for a “bedside to bench to bedside” pathophysiological identification of new drugs.

Telemedicine, biomathematical approaches and algorithmic modelling are becoming increasingly essential to follow the progression of multi-organ failure from the earliest stages and to decide a precise timing of the therapeutic intervention. For doing so, the physician should keep in mind that each drug or drug combination could have an ambivalent effect that is strictly dependent on the disease stage and the activation phase of each individual protease or in relation to all other proteases, as well as to the genetic phenotype of each individual patient.

In summary, studying early intervention with judiciously selected off-label drugs may have benefits to limit the progressive spread of SARS-CoV-2 in infected individuals. Preparation for a future for future outbreaks is a key goal and must be pursued through a better understanding of host–coronavirus interactions.

## Figures and Tables

**Table 1:** Receptors, co-receptors and proteases involved in the infection of the three recent coronavirus outbreaks.

	SARS-CoV-2	SARS-CoV	MERS-CoV
Receptor	ACE2 [10]	ACE2 [12]	DPP4 (CD26) [10]
Priming protease	TMPRSS2 [13]	TMPRSS2 [12]	
	Furin [14]	Furin [15]	TMPRSS2 [16]
	Cathepsin-L [16]	Cathepsin-L [17]	Furin [16]
	ADAM17 [17]	ADAM 17 [12]	Cathepsin-L [18]
	Factor Xa [19]	Factor Xa [20]	
Co-receptors	Sialic Acids [21]		
	NRP-1 [22]	Integrins [23]	Sialic Acids [21]
	Integrins [24]	Heparan Sulfate [25]	
	Heparan Sulfate [26]		
Other receptors	CD147 [27]	CD147 [17]	GRP78 [28]
	GRP78 [28]	GRP78 [28]	

ACE2: angiotensin-converting enzyme 2; DPP4; dipeptidyl peptidase-4; TMPRSS2: transmembrane serine protease 2; ADAM17: a disintegrin and metalloprotease 17; NRP-1: neuropilin-1; CD147: cluster of differentiation 147; GRP78: glucose regulated protein 78.

**Table 2:** Targets and mechanism of action of repurposed anti-SARS-CoV-2 drugs.

Targets	Potential Drugs	Mechanism of Action	Refs
Furin	ambroxol, bromhexine and luteolin tamarind, polyphenols, limonin and gedunin	Block the S-protein cleavage activation and membrane fusion	[13,46,47]
TMPRSS2	camostat, nafamostat, limonin, gedunin, otamixaban dabigatran and α-1-antitrypsin	Block S-protein cleavage mediated not only by TMPRSS2 but also by other proteases	[13,48,49,50]
ADAM17	α-1-antitrypsin	Blocks S-protein cleavage	[50]
Syncytia formation	niclosamide, trifluoperazine, serotonin reuptake inhibitors, ivermectin	Suppress the activity of TMEM16F, involved in syncytia formation	[51,52]
Cathepsin L	chloroquine, hydroxychloroquine and 8P9R	Interfere with the endosomial pathway, increasing pH	[53,54,55,56]
NRP-1	heparin, natural products and small molecules	Block C-end rule peptide on NRP-1	[57]
Heparan sulfate	heparin/HS mimetics	Preclude the interaction between HS and S protein	[58,59]
Integrins	ATN-161	Inhibits the interaction between S protein and integrins	[60]
CD147	azithromycin, cyclophilin A, doxycycline	Interfere with ligands/CD147 interaction	[61,62,63,64]
GRP78	epigallocatechin gallate, omoeriodictyol, isorhamnetin, and curcumin, berbamine, OSU-03012	Interfere with ligands/GRP78 interaction	[65,66]
Factor Xa	heparin	Blocks S protein cleavage by Factor Xa and inhibits the coagulation	[20]
Cyclo-oxygenase-2	non-steroidal anti-inflammatory drugs	Prevent inflammatory cytokine storm	[67]

TMPRSS2: transmembrane serine protease 2; ADAM17: a disintegrin and metalloprotease 17; NRP-1: neuropilin-1; CD147: cluster of differentiation 147; GRP78: glucose-regulated protein 78.
